# Impact of the Improved Patsari Biomass Stove on Urinary Polycyclic Aromatic Hydrocarbon Biomarkers and Carbon Monoxide Exposures in Rural Mexican Women

**DOI:** 10.1289/ehp.1002927

**Published:** 2011-05-27

**Authors:** Horacio Riojas-Rodriguez, Astrid Schilmann, Adriana Teresa Marron-Mares, Omar Masera, Zheng Li, Lovisa Romanoff, Andreas Sjödin, Leonora Rojas-Bracho, Larry L. Needham, Isabelle Romieu

**Affiliations:** 1 Environmental Health Department, National Institute of Public Health, Cuernavaca, Morelos, Mexico; 2 Center for Ecosystem Research, National Autonomous University of Mexico, and Interdisciplinary Group on Appropriate Rural Technology, Morelia, Michoacán, Mexico; 3 Division of Laboratory Sciences, National Center for Environmental Health, Centers for Disease Control and Prevention, Atlanta, Georgia, USA; 4 National Institute of Ecology, Mexico City, Mexico; 5 International Agency for Research on Cancer, Lyon, France

**Keywords:** biomarker of exposure, biomass smoke, carbon monoxide, improved stove, Mexico, polycyclic aromatic hydrocarbons, women

## Abstract

Background: Cooking with biomass fuels on open fires results in exposure to health-damaging pollutants such as carbon monoxide (CO), polycyclic aromatic hydrocarbons (PAHs), and particulate matter.

Objective: We compared CO exposures and urinary PAH biomarkers pre- and postintervention with an improved biomass stove, the Patsari stove.

Methods: In a subsample of 63 women participating in a randomized controlled trial in central Mexico, we measured personal CO exposure for 8 hr during the day using continuous monitors and passive samplers. In addition, first-morning urine samples obtained the next day were analyzed for monohydroxylated PAH metabolites by gas chromatography/isotope dilution/high-resolution mass spectrometry. Exposure data were collected during the use of an open fire (preintervention) and after installation of the improved stove (postintervention) for 47 women, enabling paired comparisons.

Results: Median pre- and postintervention values were 4 and 1 ppm for continuous personal CO and 3 and 1 ppm for passive sampler CO, respectively. Postintervention measurements indicated an average reduction of 42% for hydroxylated metabolites of naphthalene, fluorene, phenanthrene, and pyrene on a whole-weight concentration basis (micrograms per liter of urine), and a 34% reduction on a creatinine-adjusted basis (micrograms per gram of creatinine). Pre- and postintervention geometric mean values for 1-hydroxypyrene were 3.2 and 2.0 μg/g creatinine, respectively.

Conclusion: Use of the Patsari stove significantly reduced CO and PAH exposures in women. However, levels of many PAH biomarkers remained higher than those reported among smokers.

Household use of solid fuels is globally the most widespread source of indoor air pollution; solid fuels are widely used for cooking and home heating in developing countries. In Mexico, about 27 million people, mostly concentrated in rural indigenous communities, depend on biomass burning, particularly wood, for cooking and heating using traditional open fires. Compared with men, women and young children are affected to a greater extent because they spend more time in the kitchen or near the fire in the home (Masera et al. 2005). Exposure to biomass fuel combustion products, especially to wood smoke, has been associated with numerous adverse health outcomes, resulting in increased global mortality and burden of disease (Bruce et al. 2006; Naeher et al. 2007; Perez-Padilla et al. 2010; Torres-Duque et al. 2008). The International Agency for Research on Cancer (IARC) classified indoor emissions from household combustion of biomass fuel (mainly wood) as a probable carcinogen to humans (group 2A) on the basis of limited evidence in humans and experimental animals but strong evidence of mutagenicity due to the presence of polycyclic aromatic hydrocarbons (PAHs) (Straif et al. 2006). However, although PAHs have been recognized as important health-damaging biomass smoke pollutants, information about exposure levels is scarce.

Biomass fuels burned on open fires or traditional stoves emit a complex mixture containing thousands of chemicals present in the gaseous and/or particle phase of the smoke. Fine particulate matter (PM) [≤ 2.5 μm in aerodynamic diameter (PM_2.5_)] is considered the best predictor of adverse health impacts associated with biomass smoke exposures (Naeher et al. 2007), but carbon monoxide (CO), an abundant gaseous health-damaging pollutant, is easier to measure. PM and CO are the pollutants that have been most frequently quantified as indicators of personal exposure to indoor air pollution (Smith et al. 2010). However, there is little information on how representative these indicator pollutants are of other health-damaging constituents of the smoke, for example, PAHs (Torres-Duque et al. 2008). Active PM or CO monitors are effective for accessing personal exposures, but they are expensive and difficult to implement on a large scale. Color stain diffusion tubes, originally designed for industrial hygiene applications, have been suggested as an easy, less expensive, and suitable alternative for large-scale use to assess personal CO exposures (Naeher et al. 2001; Smith et al. 2010).

An approach to reduce exposure misclassification is biomonitoring. Levels of biomarkers represent the absorbed dose of a chemical, integrated across all microenvironments and routes of exposure. Some biomarkers of exposure have been evaluated as metrics of biomass smoke exposures, for example, carboxyhemoglobin, exhaled CO, urinary methoxyphenol metabolites (Clark et al. 2007), and urinary PAH metabolites (Simpson and Naeher 2010; Torres-Dosal et al. 2008).

PAHs constitute a class of compounds composed of fused benzenoid rings and, like other pollutants from biomass combustion, are formed by the incomplete combustion of organic material. Low-molecular-weight two- and three-ring PAHs are mainly in the gas phase; four-ringed PAHs are in both gas and particle phase; and high-molecular-weight five- and six-ringed PAHs are mainly associated with particles (Bostrom et al. 2002). Urinary monohydroxylated PAHs (OH-PAHs) have been used as biomarkers to assess recent exposure to PAHs, with 1-hydroxypyrene (1-PYR) the most commonly used biomarker. Concentrations of these metabolites reflect exposure to PAHs within the previous few days. The half-life for urinary 1-PYR in humans has been reported to be in the range of 4.4–35 hr (Li et al. 2009). Information on half-lives of the other OH-PAHs is scarce; however, because these are the same group of metabolites formed under similar biological pathways, we can assume that other OH-PAHs would have similar half-lives. For nonoccupationally exposed populations, diet, ambient air, tobacco smoke, and coal-tar–containing medications are the main sources of PAH exposures [Centers for Disease Control and Prevention (CDC) 2005; Naeher et al. 2007; Torres-Duque et al. 2008]. The application of biomarkers in large population studies is limited due to the resources needed for biomonitoring assays and because of issues derived from following adequate protocols for obtaining and handling biological samples limit.

One approach to reduce the health burden related to biomass fuel use has been the provision of improved wood-burning stoves. The Patsari stove is an efficient multipot wood-burning cooking stove developed with a participatory approach to meet cooking needs, which has been disseminated in rural Mexico. This stove is built in place using a mold and locally available materials; the stove includes a big flat pan or *comal*, two secondary pots, and a chimney to lead the smoke to the outside. The size and inner dimensions of the stove were optimized for a more efficient combustion (Masera et al. 2007). The Patsari stove, compared with open fire, reduces median PM_2.5_ concentrations by 71% near the stove and 58% in the kitchen (Zuk et al. 2007) and reduces mean greenhouse gases emissions by 74% (Johnson et al. 2008) and mean fuel consumption by 56% (Berrueta et al. 2007).

The aim of this study was to evaluate rural women’s exposure to CO and PAHs before and after installation of Patsari stoves. The study was performed using a small subsample because active personal exposure CO monitors and biomonitoring are costly and labor intensive, which limits their use for large-scale open population studies. An additional objective was to explore whether color-stain diffusion CO tubes could be used as a proxy measure of exposures to biomass burning pollutant emissions, such as PAHs, in future large-scale studies.

## Materials and Methods

*Study subjects and design.* The main project, titled “Improved Biomass Stove Intervention in Rural Mexico: Impact on the Respiratory Health of Women” (Romieu et al. 2009), a randomized controlled trial, was conducted during 2005 and 2006 in six communities in the Purepecha region in the highlands of Michoacán in central Mexico. Households that included a child < 5 years of age and used open wood fires for cooking (inclusion criteria) were identified by a survey. Households that agreed to participate (*n* = 668) were randomly selected by lottery drawing within each community to receive the Patsari wood cook stove at the beginning (intervention group) or at the end of follow-up (control group). A general-purpose questionnaire was administered at baseline, and the households were followed with monthly visits over a 10-month period (Romieu et al. 2009).

For the exposure biomarker evaluation study, which was performed during June–July 2005 and March–May 2006, we selected a subsample of 63 women from the Tanaco community (43 from the intervention and 20 from the control group). Tanaco is located at 2,140 m above sea level, and the average ambient temperature is 19°C (range, 8–32°C), with 48% relative humidity (range, 28–71%). There were no statistically significant differences between the women in the intervention and control groups. The participants received detailed information on the study requirements and provided written informed consent. The protocol was approved by the Bioethical and Biosecurity Committees of the Instituto Nacional de Salud Pública.

The exposure biomarker evaluation study was divided into three stages. In the preintervention stage, personal CO exposures were measured during the day while the women used a traditional open fire, and the first morning urine sample was obtained the next day to assess PAH biomarkers. During the intervention stage, a Patsari stove was installed in each participant’s home. During the postintervention stage, which took place 10 months after the Patsari stove was installed for the intervention group and 1 month after installation for the control group, CO and PAH measurements were repeated. Pre- and postintervention stages occurred in a similar season during the warmer months in this region. Information obtained during the follow-up visits for the main study indicated that some households did not fully adhere to the intervention (use of the Patsari stove) but continued using the open wood fire; these subjects were excluded in the postintervention stage of this study, leaving a total of 47 women (30 intervention and 17 controls) with complete pre- and postintervention data.

*CO exposure assessment.* We estimated personal CO exposures based on real-time measurements (continuous monitor) and cumulative samples (with diffusion tubes). We used continuous data-logging electrochemical CO monitors (Draeger Pac™III), with Draeger XS R CO sensors and a model D3T filter as supplied by the manufacturer (Draeger Safety Inc., Pittsburgh, PA, USA). The monitor was programmed to record CO in parts per million at 1-min intervals throughout the workday and was previously calibrated using CO concentrations of 50 ppm. We also used CO color-stain passive-diffusion tubes, with a detection range of 0–400 ppm-hr (CO Dositube; Gastec Inc., Kanagawa, Japan). The Draeger monitor and the passive diffusion tubes were attached to the clothing in the chest area of the women in the morning, before they started their daily activities in the kitchen. The average sampling time was 8 hr (range, 6–9.5 hr) for both stages. After the sampling time, the tubes were removed, capped, and read immediately by three different field technicians. Field technicians were trained to read the tubes using a standardized protocol, because reading has been reported to be the greatest source of error of this method (Harper 2001). The length of the stain in millimeters (range, 5–32 mm) was measured by each technician and converted to a cumulative exposure (parts per million-hour) according to a nonlinear calibration curve (*y* = 0.522*x*^1.7209^), as done in a similar study in Guatemala (Smith et al. 2010). The average CO concentration from the three readings was divided by the sampling time to determine the passive sampler CO concentration (parts per million-hour). In addition, field technicians used a time–activity questionnaire to record the activities of the women outside and inside the kitchen at 15-min intervals during sampling.

*Urine collection, PAH analysis, and exposure questionnaire.* Containers were provided to the women to collect first morning urine samples, and they were instructed on sample collection procedures. Field workers visited each home to retrieve the urine samples and conduct a brief interview to obtain information about medication, tobacco smoke exposure, and cooking practices on the previous day. A food intake questionnaire was also administered that included questions about smoked and grilled meals (fish or meat) and coffee consumed during the previous day.

Urine samples (~ 100 mL) were refrigerated at 4°C during transportation to the field office (4–6 hr) and were then frozen at –70°C. These samples remained frozen at the Instituto Nacional de Salud Pública until they were sent to the CDC for analysis. Urine samples were analyzed for OH-PAH metabolites (Li et al. 2006). The measurements of urinary PAH metabolites were subjected to a series of quality control and quality assurance checks as described by Li et al. (2006). Creatinine concentrations were determined at the CDC by an enzymatic *in vitro* assay, using clinical chemistry analyzers (Creatinine Plus; Roche Diagnostics, Indianapolis, IN, USA). Metabolites were reported as fresh-weight concentrations (micrograms per liter of urine) and as creatinine-adjusted concentrations (micrograms per gram of creatinine) to correct for urine concentration/dilution in spot samples (Barr et al. 2005).

*Statistical analyses.* We calculated geometric means (GMs) and percentiles for the biomarker levels and personal exposure measurements. To compare the subjects included and excluded from the analysis, we performed nonparametric tests on the equality of  medians. Paired comparisons of pre- and postintervention results were performed using the Wilcoxon matched-pairs signed-rank test for continuous variables, and the McNemar chi-square test for categorical variables. Spearman rank correlation coefficients between urinary PAH biomarkers and CO personal exposure measurements were estimated. We constructed scatter plots displaying the locally weighted regression (lowess) of the PAH metabolites on the CO measurements. A significance level of 0.05 was specified. Analyses were conducted using Stata software, version 9.2 (StataCorp, College Station, TX, USA).

## Results

Women from intervention and control groups had similar characteristics (Table 1). Their median age was 28 years (range, 18–44 years). Usually, families ate in the same area where the women cooked (89%), and kitchen areas were physically separated from the rest of the house (98%). Almost all kitchens had a corrugated metal roof (96%), and > 50% of the kitchens had dirt floors (66%; Table 1). Postintervention samples were available for paired comparison from 47 women (75% of the total sample: 30 intervention and 17 controls). The 16 women we excluded from the postintervention stage because of noncompliance (25% of the total sample: 13 intervention and 3 controls had age and education characteristics similar to those who completed the study but were more likely to live in a crowded household and spent more time close to the stove (data not shown). We found no significant differences in preintervention CO and PAHs exposures for these 16 women compared with the 47 subjects that completed the study.

**Table 1:** Characteristics of participating women (*n* = 47).

Characteristic	All (*n* = 47)	Intervention (*n* = 30)	Control (*n* = 17)
Women						
Age, years [median (range)]		28 (18–44)		29 (18–44)		25 (18–42)
Education, years [median (range)]		3 (0–9)		3 (0–9)		5 (0–9)
Occupation [*n* (%)]						
Housewife only		35 (74)		21 (69)		14 (78)
Housewife and another occupation						
Craftswoman		4 (9)		3 (10)		1 (6)
Seamstress		5 (11)		4 (14)		1 (6)
Trader		3 (6)		2 (7)		1 (6)
Received money from relatives in the USA [*n* (%)]		5 (10)		2 (7)		3 (17)
Household						
Persons per household [median (range)]		6 (3–11)		6 (3–11)		6 (4–10)
Crowding index, persons/room [median (range)]		1.3 (0.4–2.3)		1.3 (0.5–2.3)		1.3 (0.4–2.3)
Family eats in the same area where the woman cooks [*n* (%)]		42 (89)		27 (93)		15 (83)
Kitchen is separated from the rest of the house [*n* (%)]		46 (98)		29 (100)		17 (94)
No. of windows and open spaces between wall and roof in kitchen [*n* (%)]						
0		14 (30)		7 (28)		5 (33)
1		10 (21)		5 (20)		4 (27)
2		10 (21)		7 (28)		2 (13)
≥ 3		13 (28)		6 (24)		4 (27)
Kitchen roof type [*n* (%)]						
Corrugated metal		45 (96)		28 (93)		17 (100)
Tile		2 (4)		2 (7)		0 (0)
Kitchen floor type [*n* (%)]						
Dirt		31 (66)		21 (70)		10 (59)
Concrete		16 (34)		9 (30)		7 (41)
Kitchen volume (assuming a cubic form), m^3^ [median (range)]		46 (17–100)		48 (17–100)		41 (23–64)
Cooking practice						
Wood fuel type [*n* (%)]						
Mainly oak		24 (51)		18 (60)		7 (39)
Mainly pine		1 (2)		1 (3)		0 (0)
Combined oak and pine		22 (47)		11 (37)		11 (61)
Time spent, hours [median (range)]						
In the kitchen		4.3 (1.4–8.0)		4.5 (2.1–8.0)		4.1 (1.4–7.0)
Preparing tortillas		1.0 (0.3–3.0)		1.0 (0.3–3.0)		1.0 (0.3–2.0)
Preparing meals		2.0 (0.3–3.3)		2.0 (0.3–3.3)		1.6 (0.5–3.3)
Frying food		1.0 (0.2–3.3)		1.0 (0.2–3.3)		1.1 (0.3–2.3)

*Cooking practices and other variables.*  On average, the women spent 4.5 hr in the kitchen during the preintervention stage (open fire) and 5 hr during the postintervention stage (Patsari stove). The time spent for the different cooking tasks using an open fire (presented for the preintervention stage in Table 1) was similar to that in the postintervention stage for using the new Patsari stove (data not shown). We also collected information on other potential exposure sources to PAHs. Only one woman had a family member who smoked, and she reported being exposed to the second-hand smoke for < 1 hr/day. About one-third of the women drank coffee, but < 5% ate smoked or grilled foods (e.g., meat and fish) during the previous day. We found no significant differences before and after the intervention with respect to consumption of these foods. We developed linear regression models to estimate the effect of food intake variables on PAH biomarkers but found no significant associations (data not shown).

*Personal CO exposure measurements.* Personal exposure to CO was measured using continuous monitors and diffusion tubes (Table 2). The coefficient of variation of three readings of the tubes was 150% for preintervention and 100% for postintervention measurements. Continuous CO measurements were available for all participating women (*n* = 47), but valid CO passive samples were available for only 30 women.

**Table 2:** Personal exposure to CO concentrations for pre- and postintervention stages.

Percentile of distribution											Percent median reduction (*p*-value)*a*
CO personal exposure	Stage	5th	25th	50th	75th	90th	95th
Average of continuous sampler, ppm (*n* = 47)		Pre		1		3		4		6		9		10		75 (< 0.001)
		Post		0		0		1		2		4		5		
Maximum of continuous sampler, ppm (*n* = 47)		Pre		14		19		29		38		65		81		28 (0.056)
		Post		1		10		21		32		65		89		
Passive sampler, ppm-hr (*n* = 30)		Pre		1		2		3		5		6		10		66 (0.024)
		Post		0		1		1		4		5		6		
**a***p*-Value for Wilcoxon matched-pairs signed rank test.																

For continuous data, we estimated average and maximum values for each sample. Summary statistics were then calculated for all samples. After the installation of the Patsari stoves, personal CO exposures using continuous monitors showed a 75% reduction in average median values and a 28% reduction in average maximum values. The intervention appeared to have a greater effect on the lower end of the exposure distribution (100% reductions at the 5th and 25th percentiles) than on the upper end of the distribution (50% reduction at the 95th percentile). Postintervention CO exposures based on personal passive samplers indicated a 66% reduction in the average median concentration. The correlation between continuous and passive average personal CO exposures was higher for the preintervention stage (Spearman rho = 0.57; *p* < 0.01) than for the postintervention stage (Spearman rho = 0.42; *p* = 0.02). The preintervention samples presented a broader range of CO concentrations than did samples from the postintervention stage.

*PAH urinary biomarkers.* The World Health Organization (1996) recommends that creatinine concentration in the range of 30–300 mg/dL be considered a representative sample, that is, not too dilute or too concentrated. In our case, the creatinine concentrations ranged from 36 to 304 mg/dL,  and we included all samples in the statistical evaluation of the data. We estimated parametric statistics for 10 analytes for which the frequency of detection was ≥ 60% (Barr et al. 2004), including metabolites of naphthalene, fluorene, phenanthrene, and pyrene [for creatinine-adjusted concentrations, see Table 3; for whole-weight concentrations, see Supplemental Material, Table 1 (http://dx.doi.org/10.1289/ehp.1002927)]. Nine urinary metabolites of high-molecular-weight PAHs (benzo[*c*]phenanthrene, benz[*a*]anthracene, chrysene, and benzo[*a*]pyrene) had a low detection frequency (0–23%) and were excluded from further statistical analysis.

**Table 3:** Urinary OH‑PAH biomarkers for pre- and postintervention stages (*n* = 47).

GM (95% CI), μg/g creatinine	Percentile of distribution				Percent median reduction (*p*-value)*a*
	Metabolite	Stage	50th	75th	90th	95th
1-Hydroxypyrene (1‑PYR)	Pre	3.18 (2.63–3.85)		3.51	4.94	5.93	6.83		36 (0.001)
	Post	2.03 (1.62–2.53)		2.37	3.37	4.38	5.14		
1-Hydroxynaphthalene (1-NAP)	Pre	19.3 (15.9–23.4)		20.1	33.3	41.8	46.4		48 (0.002)
	Post	10.0 (7.36–13.6)		14.4	20.9	29.8	35.2		
2-Hydroxynaphthalene (2-NAP)	Pre	14.9 (13.2–16.8)		15.9	19.6	24.2	26.4		31 (< 0.001)
	Post	10.3 (8.74–12.2)		10.8	14.9	20.0	24.1		
2-Hydroxyfluorene (2-FLUO)	Pre	2.67 (2.37–3.01)		2.69	3.51	4.43	4.96		23 (0.029)
	Post	2.06 (1.74–2.44)		2.21	3.03	3.86	4.49		
3-Hydroxyfluorene (3-FLUO)	Pre	1.19 (1.03–1.38)		1.33	1.73	2.05	2.36		36 (< 0.001)
	Post	0.762 (0.631–0.920)		0.834	1.18	1.51	1.81		
9-Hydroxyfluorene (9-FLUO)	Pre	3.79 (3.35–4.29)		3.89	4.94	6.58	6.73		20 (0.063)
	Post	3.02 (2.53- 3.60)		3.22	4.47	5.96	7.37		
1-Hydroxyphenanthrene (1-PHEN)	Pre	2.50 (2.13–2.94)		2.53	3.30	5.56	6.96		28 (0.030)
	Post	1.81 (1.45–2.24)		2.28	2.69	3.89	4.10		
2-Hydroxyphenanthrene (2-PHEN)	Pre	1.23 (1.06–1.42)		1.18	1.59	2.22	2.49		20 (0.182)
	Post	0.984 (0.819–1.18)		1.13	1.51	2.04	2.26		
3-Hydroxyphenanthrene (3-PHEN)	Pre	1.48 (1.25–1.74)		1.44	2.29	2.71	3.19		30 (0.003)
	Post	1.03 (0.857–1.24)		1.16	1.49	2.11	2.27		
4-Hydroxyphenanthrene (4-PHEN)	Pre	0.491 (0.422–0.573)		0.459	0.677	0.844	0.998		30 (0.020)
	Post	0.346 (0.287–0.418)		0.368	0.517	0.713	0.910		
Sum of 10 OH‑PAH metabolites	Pre	52.5 (45.5–60.6)		56.9	75.6	91.8	108		34 (0.001)
	Post	34.6 (28.5–41.9)		37.5	50.1	67.0	89.3		
CI, confidence interval. **a***p*-Value for Wilcoxon matched-pairs signed rank test.									

OH-PAHs measured during the postintervention stage were significantly lower than those from the preintervention stage. Reductions in whole-weight concentrations ranged from 33% for 9_-_hydroxyfluorene (9-FLUO) to 57% for 1_-_hydroxynaphthalene (1-NAP), with a 42% total reduction for the sum of 10 metabolites [see Supplemental Material, Table 1 (http://dx.doi.org/10.1289/ehp.1002927)]. For creatinine-adjusted concentrations, reductions ranged from 20% (nonsignificant difference) for 9-FLUO to 48% for 1-NAP, with a 34% total reduction for the sum of 10 metabolites (Table 3).

**Figure 1:**
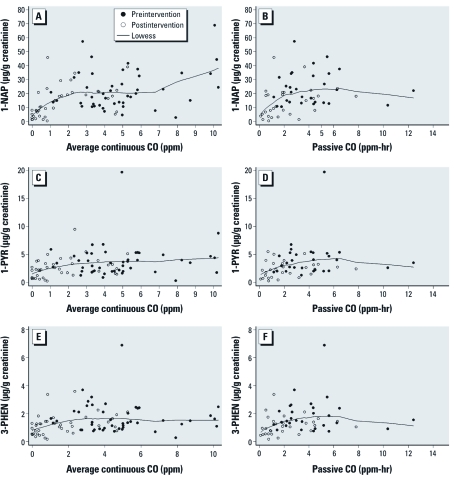
Relationship between urinary OH‑PAH biomarkers [1‑NAP (*A,B*), 1‑PYR (*C,D*), and 3‑PHEN (*E,F*)] and CO personal exposure measured by average continuous CO sampler (*A,C,E*) or passive CO sampler (*B,D,F*).

CO measurements were associated with urinary PAHs metabolites at different indoor air pollution levels (Figure 1, Table 4). In general, we found a weak association between urinary PAH metabolites and CO personal exposures during the preintervention stage (more polluted conditions), whereas a stronger, and in many cases significant, association was found during postintervention measurements (less polluted conditions). Correlations between urinary PAH metabolites and personal CO exposures measured using passive samplers were weaker than those obtained with continuous personal CO exposures.

**Table 4:** Spearman correlations for PAH urinary biomarkers (μg/g creatinine) and personal CO measures for pre- and postintervention stages (*n* = 47).

CO personal exposure	1‑PYR	1-NAP	2-NAP	2-FLUO	3-FLUO	9-FLUO	1-PHEN	2-PHEN	3-PHEN	4-PHEN
Open fire																				
Average continuous sampler		0.10 (0.509)		0.21 (0.163)		0.27 (0.070)		0.11 (0.481)		0.04 (0.768)		–0.10 (0.511)		0.04 (0.815)		0.04 (0.780)		–0.03 (0.838)		–0.13 (0.386)
Passive sampler		0.17 (0.392)		0.12 (0.535)		0.18 (0.347)		0.21 (0.285)		0.21 (0.280)		–0.30 (0.113)		0.24 (0.218)		0.02 (0.905)		0.03 (0.891)		0.02 (0.899)
Patsari stove																				
Average continuous sampler		0.29 (0.067)		0.54 (< 0.001)		0.40 (0.009)		0.49 (0.001)		0.44 (0.004)		0.17 (0.279)		0.30 (0.059)		0.38 (0.013)		0.37 (0.017)		0.28 (0.078)
Passive sampler		0.31 (0.096)		0.39 (0.037)		0.37 (0.046)		0.45 (0.012)		0.42 (0.020)		0.28 (0.136)		0.32 (0.093)		0.39 (0.033)		0.38 (0.040)		0.37 (0.048)
*p*-Values for Spearman rho are shown in parentheses.																				

## Discussion

The introduction of an improved biomass stove significantly reduced personal exposures to CO and PAHs measured as the urinary metabolites of several parent compounds. Associations between CO and two- to four-ringed PAHs varied in our study population. We observed stronger associations between CO exposures and PAH biomarker levels in postintervention relative to preintervention measurements. These results could be due to different combustion conditions and efficiency, which are highly variable for open fires. Conversely, with the Patsari stove, which was optimized in its design for more efficient combustion, we observed less variable combustion conditions. Particle size distribution in emissions has been shown to differ between Patsari stoves and open fires (Armendáriz-Arnez et al. 2010).

CO personal exposures showed significant reductions after the improved stove was introduced. The median for personal CO continuous measurements (4 ppm for open fire and 1 ppm for Patsari) resemble results from other studies performed in similar settings. A study carried out on another subsample of our intervention study population in Michoacán, in the community of Comachuén, showed mean kitchen CO concentrations of 2.3 ppm while using an open fire, and 0.5 ppm while using a Patsari stove, based on measurements made using a semicontinuous electrochemical CO sensor (HOBO monitor; Onset Corporation Inc., Bourne, MA, USA) (Armendáriz-Arnez et al. 2008). A similar study performed in Guatemala, also using the HOBO CO monitor, reported mean CO kitchen concentrations of 3.2 ppm when an open fire was in use and 0.8 ppm after an improved stove (Plancha) was installed (Clark et al. 2007).

Our findings for personal CO measured using a passive sampler (median, 3 ppm for open fire, and 1 ppm for Patsari) also resemble mean personal passive CO concentrations reported for women in the Guatemala study: 2.8 ppm for open fire and 1.4 ppm for the improved Plancha stove (Smith et al. 2010). All the results show significant reductions in CO exposures of a similar magnitude after the introduction of improved stoves.

The CO tubes are an easy-to-use, well-accepted, and accessible method for conducting personal exposure measurements, but they are not precision devices. Reading the length of stain is the single largest source of error associated with these samplers. The National Institute for Occupational Safety and Health evaluated different detector tubes and found that the accuracy of the measurements of many of them was 25–35% of the actual value (Harper 2001).

The complexity of a quantitative assessment of PAH exposures is related to the large number of individual compounds in a given mixture and by the presence of PAHs in both the gaseous and the particulate phases. The composition of emitted PAH mixtures depends on factors such as the type of fuel and its properties and the combustion technology (Bostrom et al. 2002). Comprehensive biomarker monitoring is necessary to characterize composition of the PAH mixture emitted from a biomass combustion process using either an open fire or an improved biomass stove. Data for such emission sources are scarce, and as shown here, the median reduction after an intervention is variable for different PAH metabolites.

Although four- to six-ring particulate-phase PAHs, notably benzo[*a*]pyrene, have been classified as known or probable human carcinogens, their concentrations in air tend to be low and difficult to measure (Sobus et al. 2009), and their metabolites are difficult to detect in urine samples (Toriba and Hayakawa 2007). Consistent with this, in the present study we did not measure detectable levels of high-molecular-weight PAH metabolites (benzo[*c*]phenanthrene, benz[*a*]anthracene, chrysene, and benzo[*a*]pyrene).

Generally, it is difficult to assess a carcinogenic threshold from urinary metabolites. Pyrene is not classified as a carcinogen, but it is present in the mixtures of PAHs formed during incomplete combustion (IARC 2010). The urinary metabolite 1-PYR has been widely used as a representative biomarker of PAH exposures. However, a variety of factors can influence the PAH mixture composition and benzo[*a*]pyrene:pyrene ratio; hence, a health-based exposure limit has not been determined (Jongeneelen 2004).

It was not possible to directly analyze PAH exposure routes in this study because we did not measure PAHs in air or food. However, there are no other important exposure sources of PAH exposure in ambient air for this study population, and we found no significant associations between PAH metabolites and the consumption of coffee or smoked and grilled meat and fish based on the dietary questionnaire.

Urinary 1-PYR has been established as a biomarker to evaluate PAH exposures (CDC 2005) because pyrene is normally abundant in PAH mixtures. Exposures occur via the diet, polluted air, and cigarette smoke, as well as in some occupational settings (Hecht 2002; Jongeneelen 2001; Toriba and Hayakawa 2007).

In the present study, even after the intervention, the urinary level for 1-PYR [GM, 2,025 ng/g creatinine; 95% confidence interval (CI), 1,621–2,528] was nine times higher than the concentration at the 95th percentile for females in the National Health and Nutrition Examination Survey (218 ng/g creatinine; 95% CI, 199–247) (CDC 2005). In a study carried out in rural Mexico, Torres-Dosal et al. (2008) evaluated a three-stage risk reduction program that included removing indoor soot adhered to roofs and internal walls, paving dirt floors, and introducing an improved wood stove with a chimney that vented smoke outside of the kitchen. In general, the introduction of an improved stove helps to reduce the soot deposits on cooking utensils and on the walls and ceiling of the kitchen, which also is a source of PAH exposure. The findings of Torres-Dosal et al. (2008) for urinary 1-PYR concentrations before and after the program (6.7 and 4.8 μmol/mol creatinine, equivalent to 13.0 and 9.3 μg/g creatinine, respectively) were much higher than our results presented here. In another study, Toriba and Hayakawa (2007) reported concentrations of urinary OH-PAHs for nonsmoking taxi drivers, traffic police officers, and rural villagers in Thailand. They found higher urinary concentrations of these metabolites in rural villagers, with mean concentrations of 1-PYR (1.2 μmol/mol creatinine, corresponding to 2.3 μg/g creatinine) that were similar to the levels in our study population. High urinary levels found for rural villagers were thought to be mainly associated with atmospheric PAHs emitted by open burning practices for agricultural purposes and with biomass (wood and charcoal) burning for cooking. Hecht (2002) reviewed studies reporting urinary 1-PYR levels for smokers in some European countries (mean levels < 1.0 μmol/mol creatinine, corresponding to 1.9 μg/g creatinine). Our results for rural women in the postintervention stage were on average higher than those reported in these studies for smokers.

Recently, attention has focused upon the more abundant gas-phase PAHs, notably naphthalene (two rings) and phenanthrene (three rings), as possible surrogates for PAH exposures (Sobus et al. 2009; Toriba and Hayakawa 2007). Generally, naphthalene, phenanthrene, and fluorene are the most abundant PAHs in the gaseous phase. Naphthalene has been classified as possibly carcinogenic to humans (group 2B), in contrast with phenanthrene and fluorene, which are not classifiable for carcinogenicity to humans (group 3) (IARC 2002, 2010). In the present study, we found the highest urine PAH metabolite concentrations for naphthalene metabolites, urinary 1-NAP and 2-NAP. In addition, we observed the largest percentage of reduction after the intervention for 1-NAP (57% for fresh weight, and 48% for creatinine-adjusted concentrations). After the Patsari stove intervention, the GMs for both naphthalene metabolites were between the 75th and the 90th percentile for the U.S. general female population (CDC 2005; Li et al. 2008). Toriba and Hayakawa (2007) reported that urinary naphthalene metabolites were higher among rural villagers than among taxi drivers and traffic police officers in Thailand (7.6 and 12.1 μmol/mol creatinine, corresponding to 9.7 and 15.5 μg/g creatinine for 1-NAP and 2-NAP, respectively). Our results for rural women were similar to those reported for rural villagers but higher than urinary 2-NAP levels reported for smokers in Korea (3.9 μmol/mol, corresponding to 5.0 μg/g creatinine) (Hecht 2002).

The postintervention GM for 2-hydroxyfluorene (2-FLUO) was similar to the 95th percentile reported for U.S. women (1,890 ng/g creatinine; 95% CI, 1,590–2,290), and 3-hydroxyfluorene (3-FLUO) levels were between the 90th (777 ng/g creatinine; 95% CI, 604–888) and 95th (1,030 ng/g creatinine; 95% CI, 923–1,270) percentiles for the U.S. general female population (CDC 2005).

The concentration of phenanthrene metabolites [1-hydroxyphenanthrene (1-PHEN), 186 ng/L; 2-PHEN, 164 ng/L; 3-PHEN, 162 ng/L] were much lower in firefighters responding to the World Trade Center fires and collapse (Edelman et al. 2003) than the concentrations in our study population, even in the postintervention phase. Urinary 3-PHEN levels reported for smokers in Germany (473 ng/g creatinine) (Hecht 2002) were also lower than those reported for the women participating in this study. Postintervention GMs for urinary phenanthrene metabolites were higher than the 95th percentile for the U.S. general female population (GMs, 1-PHEN, 473 ng/g creatinine; 2-PHEN, 213 ng/g creatinine; 3-PHEN, 410 ng/g creatinine; 4-PHEN, 418 ng/g creatinine) (CDC 2005).

## Conclusion

Our results show that the improved Patsari stove significantly decreases CO and PAH exposures. However, our results do not indicate that personal CO measurement is a good surrogate for measuring PAH exposure, particularly in high-pollution conditions. The reductions in CO exposure we observed following the Patsari intervention were similar to exposure reductions reported for other cook-stove intervention studies in similar populations. The CO passive sampler (color stain tube) is an easy-to-use and accessible indicator of personal CO and biomass smoke exposure but is not effective in determining specific constituents of biomass smoke such as PAHs. Even after the intervention, the levels of many PAH biomarkers were above those reported among smokers and in some occupationally exposed populations (e.g., taxi drivers, firefighters). Evidence of high postintervention levels of PAH metabolites suggests that inhabitants of this region are exposed to high levels of biomass burning emissions, even after improved stoves are installed in their homes. This may be due in part to frequent thermal inversions in the morning hours in this geographic area, which trap wood smoke until the sun heats up the lower atmosphere and the inversion is broken. Second, the Patsari stove has a flue that vents smoke outside the kitchen, and the indoor air concentrations may reflect fugitive emissions that reenter homes from outdoors. Actually, in a study conducted in this region to assess the impact of improved wood-burning stoves on PM_2.5_, Zuk et al. (2007) found similar PM_2.5_ levels in the patio before and after the Patsari stoves were installed (Wilcoxon’s test, *p* ~ 0.53).

More research is needed to advance the development of combustion-efficient improved wood stoves and further reduce pollutant emissions. Also, the long-term success and full adoption of intervention programs depend on the active participation of women from the communities, who represent the most affected population group and are most likely to benefit from improved wood stoves.

## Supplemental Material

(80 KB) PDF

## References

[r1] Armendáriz-Arnez C, Edwards RD, Johnson M, Rosas IA, Espinosa F, Masera OR (2010). Indoor particle size distributions in homes with open fires and improved Patsari cook stoves.. Atmos Environ.

[r2] Armendáriz-Arnez C, Edwards RD, Johnson M, Zuk M, Rojas L, Jimenez RD (2008). Reduction in personal exposures to particulate matter and carbon monoxide as a result of the installation of a Patsari improved cook stove in Michoacan Mexico.. Indoor Air.

[r3] Barr DB, Bravo R, Weerasekera G, Caltabiano LM, Whitehead RD, Olsson AO (2004). Concentrations of dialkyl phosphate metabolites of organophosphorus pesticides in the U.S. population.. Environ Health Perspect.

[r4] Barr DB, Wilder LC, Caudill SP, Gonzalez AJ, Needham LL, Pirkle JL (2005). Urinary creatinine concentrations in the U.S. population: implications for urinary biologic monitoring measurements.. Environ Health Perspect.

[r5] Berrueta V, Edwards R, Masera O. (2007). Energy performance of woodburning cookstoves in Michoacan, Mexico.. Renewable Energy.

[r6] Bostrom CE, Gerde P, Hanberg A, Jernstrom B, Johansson C, Kyrklund T (2002). Cancer risk assessment, indicators, and guidelines for polycyclic aromatic hydrocarbons in the ambient air.. Environ Health Perspect.

[r7] Bruce N, Rehfuess E, Mehta S, Hutton G, Smith K (2006). Indoor air pollution. In: Disease Control Priorities in Developing Countries (Jamison DT, Breman JG, Measham AR, Alleyne G, Claeson M, Evans DB, et al., eds). 2nd ed.

[r8] CDC (Centers for Disease Control and Prevention) (2005). Third National Report on Human Exposure to Environmental Chemicals.

[r9] Clark M, Paulsen M, Smith KR, Canuz E, Simpson CD (2007). Urinary methoxyphenol biomarkers and woodsmoke exposure: comparisons in rural Guatemala with personal CO and kitchen CO, levoglucosan, and PM_2.5_.. Environ Sci Technol.

[r10] Edelman P, Osterloh J, Pirkle J, Caudill SP, Grainger J, Jones R (2003). Biomonitoring of chemical exposure among New York City firefighters responding to the World Trade Center fire and collapse.. Environ Health Perspect.

[r11] Harper M. (2001). Performance evaluation of on-site colorimetric air sampling techniques.. Appl Occup Environ Hyg.

[r12] Hecht SS (2002). Human urinary carcinogen metabolites: biomarkers for investigating tobacco and cancer.. Carcinogenesis.

[r13] IARC (International Agency for Research on Cancer) (2002). Some Traditional Herbal Medicines, Some Mycotoxins, Naphthalene and Styrene.. IARC Monogr Eval Carcinog Risks Hum.

[r14] IARC (International Agency for Research on Cancer) (2010). Some Non-heterocyclic Polycyclic Aromatic Hydrocarbons and Some Related Exposures.. IARC Monogr Eval Carcinog Risks Hum.

[r15] Johnson M, Edwards R, Alatorre C, Masera O. (2008). In-field greenhouse gas emissions from cookstoves in rural Mexican households.. Atmos Environ.

[r16] Jongeneelen F. (2004). Guidelines for biological monitoring of workers in aluminium production facilities for urinary 1-hydroxypyrene (1-PYRenol).. J Environ Monit.

[r17] Jongeneelen FJ (2001). Benchmark guideline for urinary 1-hydroxypyrene as biomarker of occupational exposure to polycyclic aromatic hydrocarbons.. Ann Occup Hyg.

[r18] Li Z, Romanoff LC, Lewin MD, Porter EN, Trinidad DA, Needham LL (2009). Variability of urinary concentrations of polycyclic aromatic hydrocarbon metabolite in general population and comparison of spot, first-morning, and 24-h void sampling.. J Expo Sci Environ Epidemiol.

[r19] Li Z, Romanoff LC, Trinidad DA, Hussain N, Jones RS, Porter EN (2006). Measurement of urinary monohydroxy polycyclic aromatic hydrocarbons using automated liquid-liquid extraction and gas chromatography/isotope dilution high-resolution mass spectrometry.. Anal Chem.

[r20] Li Z, Sandau CD, Romanoff LC, Caudill SP, Sjodin A, Needham LL (2008). Concentration and profile of 22 urinary polycyclic aromatic hydrocarbon metabolites in the US population.. Environ Res.

[r21] Masera O, Edwards R, Armendáriz-Arnez C, Barrueta V, Johnson M, Rojas Bracho L (2007). Impact of Patsari improved cookstoves on indoor air quality in Michoacan, Mexico.. Energy Sust Dev.

[r22] Masera OR, Diaz R, Berrueta V (2005). From cookstoves to cooking systems: the integrated program on sustainable household energy use in Mexico.. Energy Sust Dev.

[r23] Naeher LP, Brauer M, Lipsett M, Zelikoff JT, Simpson CD, Koenig JQ (2007). Woodsmoke health effects: a review.. Inhal Toxicol.

[r24] Naeher LP, Smith KR, Leaderer BP, Neufeld L, Mage DT (2001). Carbon monoxide as a tracer for assessing exposures to particulate matter in wood and gas cookstove households of highland Guatemala.. Environ Sci Technol.

[r25] Perez-Padilla R, Schilmann A, Riojas-Rodriguez H. (2010). Respiratory health effects of indoor air pollution.. Int J Tuberc Lung Dis.

[r26] Romieu I, Riojas-Rodríguez H, Marron-Mares AT, Schilmann A, Masera O (2009). Improved biomass stove intervention in rural Mexico: impact on the respiratory health of women.. Am J Respir Crit Care Med.

[r27] Simpson CD, Naeher LP (2010). Biological monitoring of wood-smoke exposure.. Inhal Toxicol.

[r28] Smith KR, McCracken JP, Thompson L, Edwards R, Shields KN, Canuz E (2010). Personal child and mother carbon monoxide exposures and kitchen levels: methods and results from a randomized trial of woodfired chimney cookstoves in Guatemala (RESPIRE).. J Expo Sci Environ Epidemiol.

[r29] Sobus JR, Waidyanatha S, McClean MD, Herrick RF, Smith TJ, Garshick E (2009). Urinary naphthalene and phenanthrene as biomarkers of occupational exposure to polycyclic aromatic hydrocarbons.. Occup Environ Med.

[r30] Straif K, Baan R, Grosse Y, Secretan B, El Ghissassi F, Cogliano V. (2006). Carcinogenicity of household solid fuel combustion and of high-temperature frying.. Lancet Oncol.

[r31] Toriba A, Hayakawa K. (2007). Biomarkers of exposure to polycyclic aromatic hydrocarbons and related compounds.. J Health Sci.

[r32] Torres-Dosal A, Pérez-Maldonado IN, Jasso-Pineda Y, Martínez Salinas RI, Alegria-Torres JA, Díaz-Barriga F (2008). Indoor air pollution in a Mexican indigenous community: evaluation of risk reduction program using biomarkers of exposure and effect.. Sci Total Environ.

[r33] Torres-Duque C, Maldonado D, Pérez-Padilla R, Ezzati M, Viegi G. (2008). Biomass fuels and respiratory diseases: a review of the evidence.. Proc Am Thorac Soc.

[r34] World Health Organization (1996). Biological Monitoring of Chemical Exposure in the Workplace. WHO/HPR/OCH/96.1.

[r35] Zuk M, Rojas L, Blanco S, Serrano P, Cruz J, Angeles F (2007). The impact of improved wood-burning stoves on fine particulate matter concentrations in rural Mexican homes.. J Expo Sci Environ Epidemiol.

