# Supplementary material for: Impact of the Improved Patsari Biomass Stove on Urinary Polycyclic Aromatic Hydrocarbon Biomarkers and Carbon Monoxide Exposures in Rural Mexican Women
Source: Environ Health Perspect. 2011 May 27;119(9):1301–7. doi: 10.1289/ehp.1002927 (PMC3230393; doi:10.1289/ehp.1002927)
Supplement: (80 KB) PDF [file ehp.1002927.s001.pdf]

Impact of the Improved Patsari Biomass Stove on Urinary Polycyclic Aromatic Hydrocarbons Biomarkers and Carbon Monoxide Exposures in Rural Mexican Women
Supplemental Material

Horacio Riojas-Rodriguez; Astrid Schilmann; Adriana Teresa Marron-Mares; Omar Masera; Zheng Li; Lovisa Romanoff; Andreas Sjödin; Leonora Rojas-Bracho; Larry L. Needham; Isabelle Romieu

Supplemental Table 1. **Urinary OH-PAH biomarkers (µg/L urine) for pre- and post-intervention stages (N=47)**

Metabolite (µg/L urine)	Stage	GM [95% CI]	Percentiles of distribution				% median reduction (p-value)
			50%	75%	90%	95%	
1-hydroxypyrene (1-PYR)	Pre	4.30 (3.43-5.40)	4.23	7.76	11.0	13.4	47 (<0.001)
	Post	2.27 (1.82-28.3)	2.26	4.44	5.66	5.91	
1-hydroxynaphthalene (1-NAP)	Pre	25.9 (20.4-32.8)	24.2	44.3	69.7	80.9	57 (<0.001)
	Post	11.0 (8.14-14.9)	13.7	23.3	32.4	43.1	
2-hydroxynaphthalene (2-NAP)	Pre	20.1 (17.0-23.9)	20.3	31.6	45.0	54.9	43 (<0.001)
	Post	11.6 (9.62-13.9)	13.3	19.3	23.7	24.6	
2-hydroxyfluorene (2-FLUO)	Pre	3.62 (2.99-4.37)	3.74	6.11	8.46	8.88	36 (0.001)
	Post	2.33 (1.94-2.80)	2.54	3.75	4.87	5.95	
3-hydroxyfluorene (3-FLUO)	Pre	1.61 (1.32-1.97)	1.60	2.65	3.96	4.04	46 (<0.001)
	Post	0.863 (0.708-1.05)	0.883	1.37	2.13	2.67	
9-hydroxyfluorene (9-FLUO)	Pre	5.13 (4.36-6.04)	5.05	7.02	10.8	11.7	33 (0.004)
	Post	3.42 (2.81-4.15)	3.59	6.01	7.43	8.44	
1-hydroxyphenanthrene (1-PHEN)	Pre	3.39 (2.79-4.11)	3.98	5.64	7.71	8.73	40 (<0.001)
	Post	2.03 (1.62-2.53)	2.45	3.67	4.50	5.29	

2-hydroxyphenanthrene (2-PHEN)	Pre	1.66 (1.37-2.01)	1.66	2.62	3.69	4.34	33 (0.007)
	Post	1.11 (0.92-1.35)	1.20	1.76	2.74	2.80	
3-hydroxyphenanthrene (3-PHEN)	Pre	2.00 (1.62-2.46)	2.03	3.31	4.99	5.27	42 (<0.001)
	Post	1.17 (0.959-1.42)	1.20	2.11	2.73	2.99	
4-hydroxyphenanthrene (4-PHEN)	Pre	0.665 (0.555-0.797)	0.660	1.07	1.49	1.60	42 (<0.001)
	Post	0.388 (0.318-0.474)	0.455	0.642	0.829	0.928	
Sum of ten OH-PAH metabolites	Pre	70.2 (57.9-85.2)	65.9	115	166	188	42 (<0.001)
	Post	38.1 (31.2-46.5)	37.8	65.6	88.1	93.8	

GM: Geometric mean; CI: Confidence interval
P-value for Wilcoxon matched-pairs signed rank test indicated in parenthesis
